# CRISPR/Cas systems: Delivery and application in gene therapy

**DOI:** 10.3389/fbioe.2022.942325

**Published:** 2022-11-22

**Authors:** Jie Huang, Yitong Zhou, Jie Li, Aiping Lu, Chao Liang

**Affiliations:** ^1^ Department of Biology, School of Life Sciences, Southern University of Science and Technology, Shenzhen, China; ^2^ Department of Laboratory Medicine, Peking University Shenzhen Hospital, Shenzhen, China; ^3^ Institute of Integrated Bioinfomedicine and Translational Science (IBTS), School of Chinese Medicine, Hong Kong Baptist University, Hong Kong SAR, China; ^4^ Institute of Arthritis Research in Integrative Medicine, Shanghai Academy of Traditional Chinese Medicine, Shanghai, China; ^5^ Guangdong-Hong Kong-Macau Joint Lab on Chinese Medicine and Immune Disease Research, Guangzhou, China; ^6^ State Key Laboratory of Proteomics, National Center for Protein Sciences (Beijing), Beijing Institute of Lifeomics, Beijing, China

**Keywords:** CRISPR/Cas systems, delivery, viral vectors, non-viral vectors, genome editing, gene therapy

## Abstract

The CRISPR/Cas systems in prokaryotes such as bacteria and archaea are the adaptive immune system to prevent infection from viruses, phages, or other foreign substances. When viruses or phages first invade the bacteria, Cas proteins recognize and cut the DNA from viruses or phages into short fragments that will be integrated into the CRISPR array. Once bacteria are invaded again, the modified CRISPR and Cas proteins react quickly to cut DNA at the specified target location, protecting the host. Due to its high efficiency, versatility, and simplicity, the CRISPR/Cas system has become one of the most popular gene editing technologies. In this review, we briefly introduce the CRISPR/Cas systems, focus on several delivery methods including physical delivery, viral vector delivery, and non-viral vector delivery, and the applications of disease therapy. Finally, some problems in CRISPR/Cas9 technology have been proposed, such as the off-target effects, the efficiency of DNA repair mechanisms, and delivery of CRISPR/Cas system safely and efficiently to the target location.

## Introduction

The disease is the common enemy of human mankind. Many biological scientists and clinicians have developed and innovated a variety of emerging therapeutic tools and strategies for different diseases such as gene therapy. This therapy modifying genes to treat or prevent diseases mainly target genetic diseases, some cancers, and viral infections. Despite being an emerging therapy, gene therapy exerts an important function in clinical application such as new drug development. Many gene therapy drugs, such as Zolgensma, Strimvelis, Luxturna, LentiGlobin, etc, are produced after the modification of gene editing tools (zinc finger nucleases (ZFNs), transcription activator-like effector nuclease (TALENs), and clustered regularly interspaced short palindromic repeats (CRISPR)/CRISPR-associated (Cas) system). For gene therapy, these tools show huge potential and function.

Before the CRISPR/Cas system (also known as third-generation genome editing tool), ZFNs and TALENs are the commonly used gene-editing tools. There are some limitations in these two technologies, such as low editability, high off-target rate, high cytotoxicity, high cost, time consumption, and labor consumption. Compared with TALENs and ZFNs, the CRISPR/Cas system is simpler to design, lower cost, higher targeting efficiency, lower cost, lower off-target rate, and lower cytotoxicity. Besides, this technology can edit many different genes *in vitro* or *in vivo* (Bharathkumar et al., 2022). Based on these advantages, the CRISPR/Cas system currently has become a potent genome editing tool in the field of molecular biology. On the other hand, under the optimization trend of CRISPR/Cas 9 system cleavage elements in the future, CRISPR/Cas9-based CRISPR technology may dominate the future of gene editing despite the use more of TALENs technology in clinical practice now.

**FIGURE 1:**
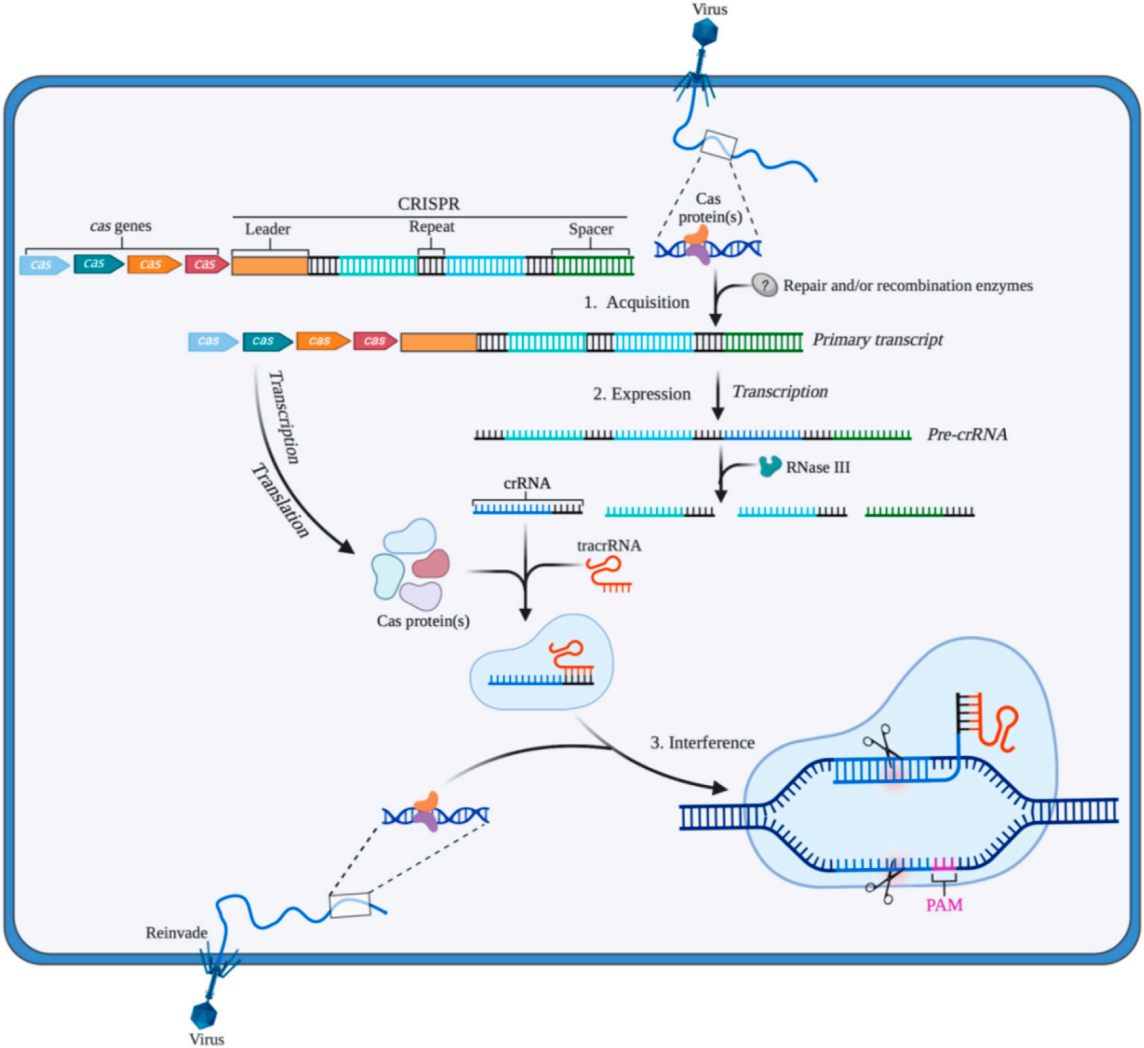
The CRISPR/Cas adaptive immune system in prokaryotes (1) Acquisition: in this stage, the invading DNA is fragmented, and then a new protospacer is selected and integrated as a new spacer in the CRISPR array; (2) Expression: during this second stage, the pre-crRNA produced by the CRISPR array is cleavaged into mature crRNAs by RNase III. The mature crRNAs, tracrRNAs, and Cas proteins assemble to form ribonucleoprotein (RNP) complexes; (3) Interference: at the final stage, the crRNP identifies invading DNA by small guide RNA (sgRNA) (crRNA:tracrRNA), and Cas protein cut the foreign DNA, thereby removing the foreign genetic materials. Created with BioRender.com.

The CRISPR/Cas system existing in prokaryotes such as bacteria and archaea is the adaptive immune system to prevent infection from viruses, phages, or other foreign substances. CRISPR, a repeating sequence discovered firstly by Ishino in the genome of *E. coli*, contains a large number of adjacent leader sequences and repeat sequences (Ishino et al., 1987). As an important component in this system, recognition is the main function of Cas proteins. Additionally, different types of Cas proteins show their specific functions in the acquisition, expression, or interference stage. When viruses or phages first invade the bacteria, Cas proteins recognize and cut the DNA from viruses or phages into short fragments that will be integrated into the CRISPR array (Makarova et al., 2011). CRISPR RNA (crRNA) and transactivated CRISPR RNA (tracrRNA) are produced by CRISPR once bacteria are invaded by the same foreign substance again. The complex containing crRNA, tracrRNA, and Cas protein is formed. Then, crRNA identifies and matches with the foreign DNA by base pairing, which guides Cas protein to cut target DNA sequences of the virus or phages to protect their hosts (Makarova et al., 2011) (Figure 1).

Since it has been shown to cut DNA *in vitro* in 2012 (Gasiunas et al., 2012; Jinek et al., 2012), CRISPR/Cas9 gene editing technology has been rapidly improved and applied in many fields such as biology, biomedicine, agriculture, environment. Note of Zhang’s team found widespread CRISPR/Cas9 system in prokaryotes can be used in eukaryotes (Cong et al., 2013). Studies about the CRISPR/Cas system in the past decade have shown the significant potential of this tool in treating diseases related to genetic mutation or change, such as Alzheimer’s disease, huntington’s disease, non-small-cell lung carcinoma (NSCLC), and cardiovascular diseases. The safety and feasibility of CRISPR/Cas 9 gene editing technology by knocking out PD-1 in T cells are proved in a clinical study treating NSCLC by Lu in 2020, but the therapeutic efficacy should be improved in the next clinic experiments (Lu et al., 2020). 38 clinical studies using CRISPR technology to treat diseases can be found at *ClinicalTrials.gov.* Until 23 September 2022. The huge strengths and potential in gene therapy make the CRISPR/Cas system more important in the biology and biomedicine field.

In this review, we briefly introduce and summary the classification, several delivery systems (physical delivery, viral vector delivery, and non-viral vector delivery), and the applications of the CRISPR/Cas system, as well as discuss the limitations and perspective of CRISPR/Cas tool for gene therapeutics.

## CRISPR/Cas systems

**FIGURE 2:**
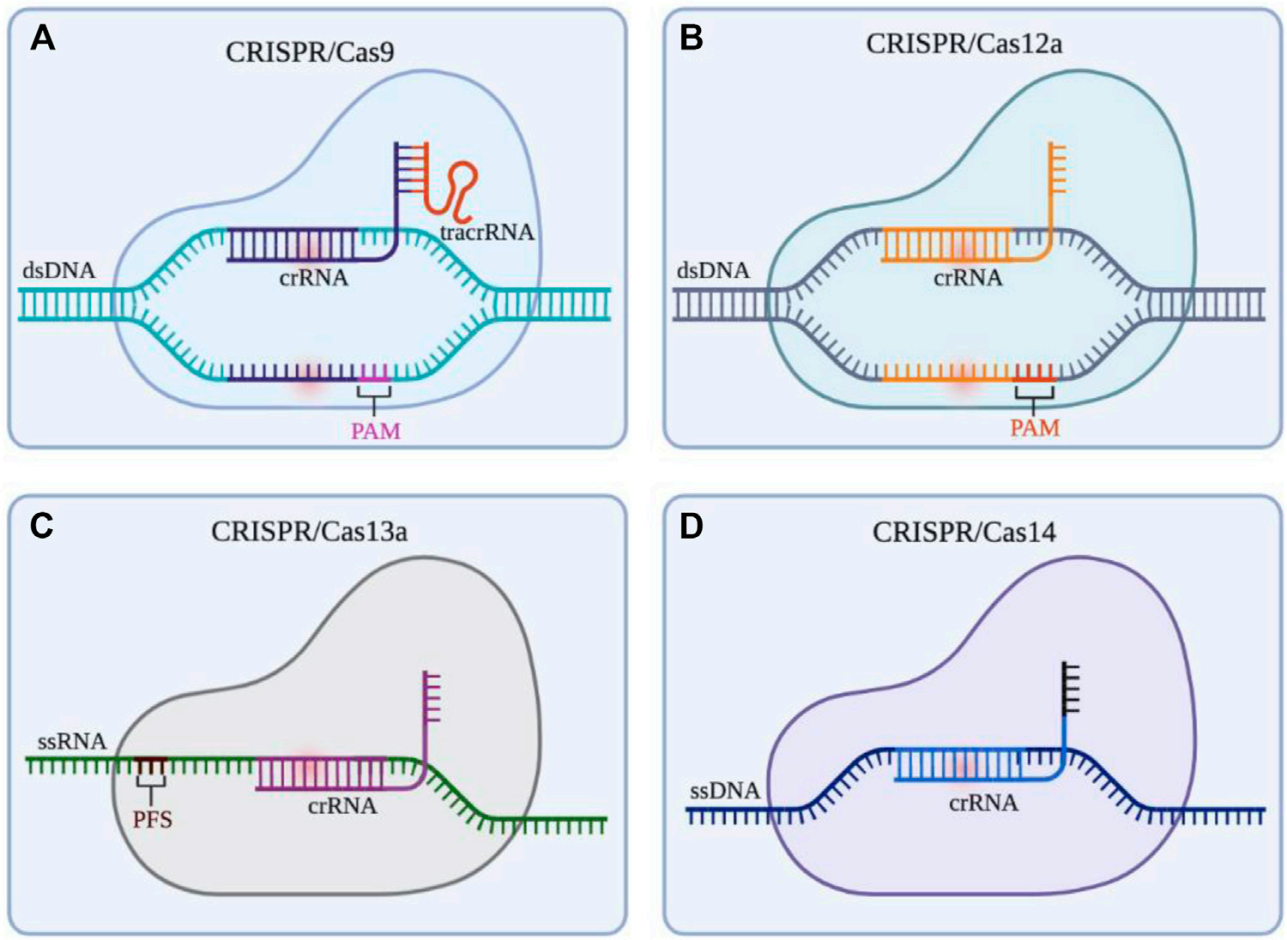
Gene editing schematic of four commonly used CRISPR/Cas systems (Cas9, Cas12a, Cas13a, and Cas14). **(A)** CRISPR/Cas9 system is able to cleave at 3bp upstream of the double stranded DNA (dsDNA) PAM by the guidance of small guide RNA (sgRNA) (crRNA: tracrRNA), resulting in the double strand break (DSB) of the target site and the blunt ends. **(B)** CRISPR/Cas12a system only depends on crRNA to recognize the PAM of dsDNA, then cleaves the target DNA in turn to produce sticky ends **(C)** CRISPR/Cas13 system utilizes crRNA to target and cleave single stranded RNA (ssRNA) downstream of the PFS. **(D)** The action mode of CRISPR/Cas14 system is similar to CRISPR/Cas12a system, but it does not rely on PAM to identity and cut single stranded DNA (ssDNA). Created with BioRender.com.

The CRISPR/Cas system can be divided into two categories according to the effect submodule organization, of which I, III, and IV are the first category (Class 1); II, V, and VI are the second category (Class 2). The Class 1 CRISPR/Cas system mainly utilizes a multi-protein effector complex to achieve nucleic acid cleavage; while in the Class 2 CRISPR/Cas system, single-protein effector, that is, only a single Cas protein nuclease such as Cas9, Cas12, Cas13, and Cas14 is required to complete the cleavage of the target (Makarova et al., 2015). The Class 1 CRISPR/Cas system is rarely applied in eukaryotic gene engineering due to the comparatively difficult heterologous expression of multiple groups of hierarchically linked complexes (Makarova et al., 2015). The Class 2 CRISPR/Cas system is widely used in basic and translational biomedical research due to the advantages of single nuclease application. As summarized in Figure 2, Class 2 CRISPR/Cas system is the common genome editing tool in gene therapy. Therefore, we focus on the CRISPR/Cas systems based on Cas9, Cas12, Cas13, and Cas14.

### CRISPR/Cas9 system

The CRISPR/Cas9 system, the most powerful and potent gene editor tool currently, is commonly and widely applied for gene modification in various cells and animals (Figure 2A). Cas9 protein containing HNH and RuvC endonuclease active sites can cleave double-strand DNA with the guidance of crRNA (Jinek et al., 2012). In this process of the CRISPR/Cas 9 System, the transactivating crRNA (tracrRNA) involves the formation the ribonucleoprotein (RNP) complex (crRNA: tracrRNA: Cas9). It is worth noting that *Streptococcus pyogenes* Cas9 (SpCas9) is the first and common Cas effector for genome engineering by identifying protospacer adjacent motif (PAM) sequences (5′-NGG-3′) (Chylinski et al., 2013; Anders et al., 2014). Homology-directed repair (HDR) and non-homologous end joining (NHEJ) will happen to repair the double strands break (DSB) after cutting DNA by Cas9. In addition, other variants of Cas9 such as *Staphylococcus aureus* Cas9 (SaCas9) broaden the range of target locus, this is because it can identify the PAM sequence including 5′-NNGRRT (NNGAAT, NNGAGT, NNGGAT, NNGGGT) (Ran et al., 2015). Besides, to solve some limitations in the system such as limited genome-targeting scope restricted by PAM sequences, off-target effect, and low efficiency and specificity, many researchers have developed various advanced systems (*i.e.,* dead-Cas9 system, base editing system, Cas9 variant system, prime editing system) (Xu and Li, 2020). Up to now, the CRISPR/Cas9 system still is the workhorse of gene editing, and its development changes and revolutionizes the range from biological engineering to biomedical applications. However, CRISPR/Cas9-mediated clinical treatment still faces many challenges and difficulties like highly efficient delivery and off-target effects.

### CRISPR/Cas12 system

Since Cas12a (known as Cpf1) was founded by Zhang’s team in 2015 (Zetsche et al., 2015), the CRISPR/Cas12 system has also been encouraged to develop genome editing technology (Figure 2B). Differing from Cas9, Cas12a recognizes 5′-TTTV-3′ PAM and generates sticky ends, which improves the increased efficiency and precision of HDR-mediated insertion (Swarts et al., 2017). More importantly, Cas12a only needs the guidance of crRNA to recognize dsDNA without the assistance of tracrRNA. Apart from that, in 2018, Chen surprisingly found Cas12a can cleave single-stranded DNA (ssDNA) with indiscrimination to reduce activity, which means viral DNA in patients’ samples can be detected by the CRISPR/Cas12a system, further providing a diagnostic tool for the clinic (Chen et al., 2018). What’s more, Cas12a can recognize and cut target genes several times after cutting, which is because the incision formed by Cas12a is far from the PAM site, and the nucleotide insertion or deletion caused by NHEJ will not lead to PAM sequence changes (Swarts et al., 2017). In clinic, CRISPR/Cas12a system also is used for detecting pathogens in pevere pneumonia and sepsis (NCT04178382, NCT05143593). In the future, the application of the CRISPR/Cas12a system might be much wider in clinical therapy. Another Cas protein, Cas12b (also known as Cpf1 C2C1) is an RNA-directed nuclease, but it has not yet been fully developed and this may be due to its high-temperature addiction. In 2018, Zhang and his colleagues redesigned Cas12b to enhance its activity at human body temperature (37°C), and the remodified Cas12b has a higher specificity for target sequences in cell culture experiments (Strecker et al., 2018). There are still many difficulties promoting the CRISPR/Cas12b system to be a mature gene editing tool.

### CRISPR/Cas13 system

As targeting CRISPR DNA enzymes, Cas9 and Cas12 bring many new possibilities to modify and manipulate DNA. Cas13, a new type of enzyme targeting RNA, was also uncovered with great efforts by researchers in the past few years. At present, there are the four known subtypes in the Cas13 family, containing Cas13a (C2c2), Cas13b (C2c4), Cas13c (C2c7), and Cas13days (CasRX). CRISPR/Cas13a system targeting RNA was first described in 2016 by Zhang’s team (Figure 2C) (Abudayyeh et al., 2016). After recognizing the target RNA by crRNA without tracrRNA and binding to this DNA, the capacity of Cas13a to collateral cleavage to the RNAs will be activated. But the collateral cleavage activity in eukaryotic species was not found and the molecular mechanism of this activity was not clear (Abudayyeh et al., 2016). Nonetheless, this type of RNA targeting tool based on CRISPR has already been applied in biomedical applications, such as detecting the specific sequences of tumor circulating RNA and viral RNA in patients (Gootenberg et al., 2017; Dincer et al., 2019). CRISPR/Cas13 systems have huge potential in treating cancer or other diseases by editing and modifying key RNA molecules (*e.i.*, mRNAs, microRNAs, lncRNAs, snoRNA) (Li et al., 2019).

### CRISPR/Cas14 system

In 2018, researchers discovered the CRISPR/Cas14 system in a group of archaea (Figure 2D). Cas14 contains a conserved RuvC nuclease domain and is about one-third the size of Cas9. Cas14 is able to target ssDNA cleavage without requirements of limiting sequences, which is highly specific to single-stranded DNA compared to Cas12a (Harrington et al., 2018). Like the Cas9 protein, Cas14 has the potential to be used as a biotechnology tool. Improvements to Cas14 have the potential to improve the CRISPR diagnostic system currently being developed for the rapid diagnosis of infectious diseases, genetic mutations, and tumors.

## CRISPR/Cas system delivery methods

The CRISPR/Ca systems, as the most popular gene editing tool, can mediate multifunctional and high-precision genome modification, realizing the treatment of a variety of major diseases such as tumors, genetic diseases, and infectious diseases. Normally, the CRISPR/Cas system needs delivery strategies including *in vitro*, *in vivo*, and *ex vivo* to exert its function in disease treatment. A very necessary prerequisite for the function of the CRISPR/Cas9 system is efficient delivery to target cells. To be more specific, the CRISPR/Cas system can be delivered into cells in three different forms (Table 1). The first form is to deliver sgRNA and mRNA of Cas9 protein, but mRNA can be translated directly in the cytoplasm, but the stability of mRNA is poor, and its rapid degradation will limit the duration of gene editing (Niu et al., 2014). The second type is to deliver plasmid DNA (pDNA) encoding Cas9 and sgRNA, pDNA is more stable than delivering mRNA but the generation efficiency of Cas9 might be low due to the necessity of pDNA entering the nucleus (Ran et al., 2013b). Delivering the Cas9/sgRNA RNP complex is the third method, this way does not require transcription and translation processes, and can initiate genome editing faster and reduce off-target effects. However, the large size of the Cas9 enzyme itself limits the delivery efficacy to a certain extent and producing large quantities of highly active Cas9 protein also is difficult (Kim et al., 2014; Zuris et al., 2015; Park and Choe, 2019).

**TABLE 1:** Three delivery strategies of CRISPR/Cas9 system.

Modes	Advantages	Disadvantages	References
Cas9 mRNA + sgRNA	Faster	Poor stability	Niu et al. (2014)
	Low off-target effect		
CRISPR/Cas9 plasmid DNA (pDNA)	Simple and high stability	Low efficiency	Ran et al. (2013b)
		Delayed onset	
		Integration risk	
Cas9: sgRNA ribonucleoprotein complex (RNP)	High editing efficiency	Difficult to package	Kim et al. (2014); Zuris et al. (2015); Park and Choe (2019)
	Low off-target effect		
	Low immunogenicity		
	Swift onset		

Delivering the CRISPR/Cas system to its target with high efficacy and precision is a complex and tough project. To improve and solve these problems mentioned above, physical delivery, viral delivery, and non-viral delivery have been utilized to send CRISPR/Cas systems to cancer cells or immune cells for manipulating critical gene.

### Physical delivery methods

The physical delivery methods relying on transient membrane disruption, include microinjection, hydrodynamic injection, electroporation, and other methods including membrane deformation, sonoporation, and lance array nanoinjection (LAN) (Table 2) (Wang et al., 2017).

**TABLE 2:** Physical delivery of CRISPR/Cas systems.

Approaches	Delivery strategies	CRISPR/Cas systems	Target genes	Target cells/tissues	Species/diseases	References
Microinjection	*in vitro*	Cas9 mRNA and sgRNAs	Ppar-γ, Rag1	One-cell embryos	Cynomolgus monkey	Niu et al. (2014)
	*in vitro*	Cas9 mRNA and sgRNAs	APOE, B2M, PRF1, and PRKDC	Fertilized eggs	Rat	Ma et al. (2014)
	*in vitro*	Cas9 mRNA and sgRNAs	Gal4	Embryos	Zebrafish	Kimura et al. (2014)
	*in vitro*	Cas9 mRNA and sgRNAs	APOE, CD36, CFTR, LDLR, apoC-III, SCARB1, LEP, LEP-R, RyR2	Embryos	Rabbits	Yang et al. (2014)
	*in vitro*	Cas9 mRNA and sgRNAs	MSTN	Zygotes	Sheep	Crispo et al. (2015)
	*in vitro*	Cas9 protein and sgRNA	-	Embryos	*Xenopus laevis*	Corkins et al. (2022)
Electroporation	*in vitro*	CRISPR/Cas9 plasmid	APP^Swe^, PSEN1^M146V^	iPS cells	Human	Paquet et al. (2016)
	*in vitro*	CRISPR/Cas9 plasmid	exon 45–55 mutation hotspot region	Myoblasts	DMD	Ousterout et al. (2015)
	*ex vivo*	CRISPR/Cas9 plasmid	PD-1	Human primary T cells	Cancer	Su et al. (2016)
	*ex vivo*	Cas9/sgRNA RNP	TGFBR2, HPRT1	Human NK Cells	-	Naeimi et al. (2018)
	*In vivo*	Cas9/sgRNA RNP	Sox2, GFP	-	Axolotl	Fei et al. (2016)
	*in vivo*	Cas9/sgRNA RNP	exon80	Skin	Mouse	Wu et al. (2017b)
Hydrodynamic injection	*in vivo*	CRISPR/Cas9 plasmid	HBV	Hepatoma cells	Transgenic mouse	Zhu et al. (2016)
	*in vivo*	CRISPR/Cas9 plasmid	Pten, p53	Liver	Mouse	Xue et al. (2014)
Membrane deformation	*in vitro*	CRISPR/Cas9 plasmid	GFP, NUAK2, *AAVS1*	293T, MCF7, SUM159, SU-DHL-1, AB2.2	Human, mouse	Han et al. (2015)
Sonoporation	*in vitro*	Cas9/sgRNA RNP	GFP	B16F10	Mouse	Hansen-Bruhn et al. (2018)
Lance array nanoinjection	*in vitro*	CRISPR/Cas9 plasmid	GFP	HeLa	Human	Sessions et al. (2016)

Notes: Ppar-γ: peroxisome proliferator-activated receptor; Rag1: recombination activating 1; APOE: apolipoprotein; B2M: beta-2-microglobulin; PRF1: perforin 1; PRKDC: protein kinase, DNA-activated, catalytic subunit; GAL4: galactose-responsive transcription factor; CD36: cluster of differentiation 36; CFTR: cystic fibrosis transmembrane conductance regulator; LDLR: low-density lipoprotein receptor; apoC-III: apolipoprotein C-III; SCARB1: scavenger receptor class B,member1; LEP: leptin; LEP-R: leptin receptor; RyR2: ryanodine receptor 2; MSTN: myostatin; iPS: pluripotent stem cells; APP^Swe^: amyloid precursor protein; PSEN1^M146V^: presenilin 1; DMD: duchenne muscular dystrophy; PD-1: programmed death-1; TGFBR2: TGF-beta type II, receptor; HPRT1: hypoxanthine phosphoribosyltransferase 1; HBV: hepatitis B virus; GFP: green fluorescent protein; RNP: ribonucleoprotein.

*Microinjection* deliver the CRISPR/Cas system into cells by glass needles, which is the most direct approach. Microinjection sends the molecular cargoes into the cytoplasm in a controlled manner without considering the barriers of the extracellular and cytoplasmic (Graessmann and Graessmann, 1983). When using this kind of method, the size and weight Cas proteins/sgRNAs do not need to be considered. By one-step injection of Cas9 mRNA and sgRNA into cells, Ma et al. and Niu et al. successfully knocked out target genes in the zygotes of rats and monocellular stage embryos of cynomolgus monkeys, respectively (Ma et al., 2014; Niu et al., 2014). Moreover, Corkins et al. also successfully microinjected sgRNA along with the into the zygote of *Xenopus laevis* frogs to disrupt the genomic sequences in the whole embryo (Corkins et al., 2022). Apart from the mentioned animal, various cells from other different animals also have been efficiently edited *via* this technique such as zebrafish, mouse, rabbit, sheep (Yang et al., 2013; Kimura et al., 2014; Yang et al., 2014; Crispo et al., 2015). Nonetheless, microinjection is a difficult strategy that can deal with two or three hundred cells for each trial. Hence, it is unreasonable to treat millions or even billions of cells at the same time despite microinjection is significant to edit single-cell genome. Additionally, this method can only be used *in vitro* since access to cells is not possible for microinjection by *in vivo*.

*Electroporation* Another popular physical method of transferring RNA/DNA into cells is electroporation. It accomplishes intracellular delivery by means of transient interruption of the lipid bilayer comprising the plasma layer. To date, CRISPR/Cas9 can be sent well *in vitro* and *in vivo* by electroporation. For example, CRISPR/Cas9 components are successfully introduced into induced pluripotent stem (iPS) cells and myoblasts by electroporation to edit target genes in the studies of Paquet et al. and Ousterout et al., respectively (Ousterout et al., 2015; Paquet et al., 2016). Besides, electroporation, contrasted with microinjection, brings about a higher embryo survival rate, which has the potential to reduce the number of animals used to produce transgenic mouse models. However, there exist some problems such as cell death and loss of cell stemness when this technique is applied (Laustsen and Bak, 2019). Researchers try to minimize the issue by adjusting and optimizing electroporation parameters and medium composition.

*Hydrodynamic injection* delivery is also one of the physical delivery methods. Some studies showed the potential of this method, for instance, Xue et al. used hydrodynamic injection to deliver CRISPR pDNA expressing Cas9 and sgRNA to the liver through tail vein injection, directly targeting tumor suppressor genes PTEN and P53, resulting in their deletion, and finally generating hepatocellular carcinoma mouse model (Xue et al., 2014). Zhen et al. injected CRISPR/Cas9 components targeting the coding region of HBsAg, which destroys HBV, into HBV-infected mice by tail vein injection. Immunohistochemical results showed that there were almost no HBsAg positive cells in the liver tissue of mice in the experimental group, which effectively generated mutations in HBV DNA, and inhibited HBV increase in HBV-infected mice (Zhen et al., 2015). However, the hydrodynamic delivery method is prone to organ trauma, resulting in potential physiological complications such as increased blood pressure and liver dilation. When used in mouse models, it is easy to cause the accidental death of mice, and the transfection efficiency is low, suitable only for some cells. Therefore, there are currently no clinical applications.

*Other physical delivery methods* Other physical delivery methods also exert an effective role in the delivery of the CRISPR/Cas system. The membrane distortion of cells can enhance the delivery of CRISPR/Cas systems into cells with low cell toxicity.

Han and others successfully transfer the plasmid encoding Cas9 and sgRNA-EGFP into cells by optimizing a microfluidic device based on membrane deformation, achieving highly efficient genome editing (N90% EGFP knockout efficiency) (Han et al., 2015). In addition, Hansen-Bruhn employed an ultrasound-powered nanomotor to deliver Cas9/sgRNA complex with just 0.6 nm, knocking out more than 80% GFP after 2 h of cell incubation, which indicates the potential promise for highly efficient therapeutic applications (Hansen-Bruhn et al., 2018). What’s more, the utility of LAN was demonstrated, Sessions successfully delivered the CRISPR-Cas9 system to edit the genome of isogenic cells by changing the serial injection method and the electrical current settings (Sessions et al., 2016).

In physical methods, DNA/mRNA/protein without any carrier is transferred into cells, leading to the enzymatic degradation and rapid clearance of naked DNA/mRNA/protein in tissues or systemic circulation. Therefore, chemical nonviral delivery as an alternative are more likely to play a predominant function in the future.

### Viral vector delivery methods

As one of the most popular delivery methods, viral vectors mainly contain adeno-associated viruses (AAVs), adenovirus (AdVs), and lentivirus (Table 3) (Xu C. L. et al., 2019). The method of virus delivery requires that HEK 293T cells be packaged to produce viral-like particles containing Cas9 and sgRNA. It then infects the target cells, which are then transported into the body or studied *in vitro*.

**TABLE 3:** The CRISPR/Cas-loaded viral vectors potential for gene therapy.

Virus	Insert capacity	Delivery strategies	Cargos	Target cells/tissues	Target genes	References
AAVs	4.7 kb	*in vivo*	CRISPR/Cas9 system	Brain	MECP2, Dnmt1, Dnmt3a, Dnmt3b	Swiech et al. (2015)
		*ex vivo*	CRISPR/Cas9 system	AML12	Apoa1	Zhan et al. (2019)
		*in vitro*	CRISPR/Cas9 system	Human primary retinal microvascular endothelial cells	VEGFR2	Wu et al. (2017a)
AdVs	7.5 kb	*in vivo*	CRISPR/Cas9 system	Lung	Eml4, Alk	Maddalo et al. (2014)
		*in vitro*	CRISPR/Cas9 system	Human primary cells	αSMA, FN1	Voets et al. (2017)
		*in vivo*	CRISPR/Cas9 system	Liver	PCSK9	Ding et al. (2014)
Lentivirus	8 kb	*in vivo*	CRISPR/Cas9 system	SMMC-7721	HIF-1*α*	Liu et al. (2018)
		*in vivo*	CRISPR/Cas9 system	SW403	KRAS	Kim et al. (2018)
Baculovirus	>38 kb	*ex vivo*	CRISPR/Cas9 system	iPS	HMGA1	Mansouri et al. (2017)
Sendai virus	5 kb	*ex vivo*	CRISPR/Cas9 system	Human primary monocytes	CCR5, EFNB2	Park et al. (2016)
Epstein-Barr virus	5 kb	*ex vivo*	Cas9/sgRNA RNP	Human primary B cell	-	Jiang et al. (2018)

Notes: AAVs: adeno-associated virus; AdVs: adenovirus; MECP2: methyl-CpG, binding protein 2; DNMT1, 3a,3b: DNA, methyltransferase 1, 3a, 3b; iPS: pluripotent stem cells; CCR5: CC, chemokine receptor 5; EFNB2: ephrin B2; RNP: ribonucleoprotein; ALK: anaplastic lymphoma kinase*;* VEGFR2: Vascular endothelial growth factor receptor-2; *EML4: echinoderm microtubule-associated protein-like 4;* αSMA: alpha-smooth muscle actin; FN1: fibronectin 1; Pcsk9: proprotein convertase subtilisin/kexin type 9; HIF-1*α:* hypoxia inducible factor-1α; KRAS: kirsten ratsarcoma viral oncogene homolog.

*AAVs* Among these viral vectors, AAVs have largely been applied for CRISPR gene editing. Due to their good safety profile and therapeutic potential, AAVs have already been approved to perform many clinical trials for gene therapy (Lau and Suh, 2017). Furthermore, the immunogenic of AAVs are significantly less than other viruses. However, AAVs are small and have strict requirements on the size of the packaged material, so researchers have proposed that Cas9 and sgRNA can be packaged separately and infect cells together. For example, Swiech et al. injected a 1:1 mixture of AAV-SpCas9 and AAV-sgRNA (targeting MecP-2 gene) into hippocampal dentate gyrus (DG) of adult male mice. At 4 weeks after virus injection, the co-transduction efficiency of the two vectors in hippocampal granulosa cells was about 80%, and the modification efficiency of the mecP-2 gene was about 70%. Other researchers have proposed that smaller Cas9 proteins can be found in different types of bacteria. Ann Ran et al. found that Cas9 from SaCas9 is more than 1 KB smaller than the commonly used Cas9 from SpCas9 (Ran et al., 2015). The authors delivered AAV-SACAS9: sgRNA targeting the cholesterol-regulating gene Pcsk9 into mice by tail vein injection, and >40% Pcsk9 gene editing was observed within 1 week after injection, and total cholesterol level of the mice decreased significantly. In addition, viral vectors may also have certain immunogenicity and risk of mutation, resulting in certain limitations in the clinical application (Follenzi et al., 2007; Ahi et al., 2011). Recently, to improve the efficiency of AAV, Tsuji et al. applied fludarabine, RNR inhibitor, in a short-term administration increased the *in vivo* efficiency of AAV- and CRISPR/Cas9-mediated homologous recombination (Tsuji et al., 2022).

*AdVs*, non-enveloped, dsDNA virus, can infect both dividing and non-dividing cells thanks to the special structure with an icosahedral nucleocapsid. With the deep development, AdVs-based CRISPR delivery systems show a potent function in the establishment of disease models, the development of tools for drug discovery, and the treatment of existing diseases. For example, Maddalo et al. induced Eml4-ALK oncogene rearrangement *in vivo* using AdV-mediated CRISPR/Cas9 system to generate an EML4-ALK gene-driven lung cancer mouse model (Maddalo et al., 2014). With regard to drug discovery, Voets et al. slienced the *SMAD3* gene in human lung fibroblasts and bronchial epithelial cells by AdV (Voets et al., 2017). In the study of Ding et al., they found the loss-of-function mutation of PCSK9 in mouse livers can reduce cholesterol levels in plasma (Ding et al., 2014).

*Lentivirus* is a single-stranded (ss) RNA spherical virus. One extraordinary benefit of the lentivirus is its capacity to be pseudotyped with other viral proteins. This takes into account the designing and adjusting of the lentivirus’ cell tropism (Lino et al., 2018). What’s more, lentivirus vectors are erased of the relative multitude of viral qualities and do not trigger the immune system (Bennett, 2003; Pauwels et al., 2009). As a retrovirus, it integrates into the host genome, which increases unwanted off-target insertional mutagenesis in CRISPR/Cas9 delivery (Pauwels et al., 2009; Lebas et al., 2017). Nevertheless, the researchers still have made some progress. To treat human hepatocellular carcinoma (HCC), Liu et al. first knocked out HIF-1α through a lentivirus-mediated CRISPR/Cas9 system with sgRNA-721 (LV-H721). Then LV-H721 was then directly injected into the tumor tissues of the subcutaneous xenograft model SMMC-7721. The level of HIF-1α in the tumor tissues after 3 days of treatment by injecting a lentivirus-mediated CRISPR/Cas9 system (Liu et al., 2018). According to Kim et al., lentivirus and AAV expressing Cas9 and sgRNA were used to target mutant KRAS alleles in cancer cells. By injecting intratumorally (*i.t.*) into colon carcinoma xenografts, tumor growth was inhibited effectively (Kim et al., 2018).

### Non-viral vector delivery methods

**FIGURE 3:**
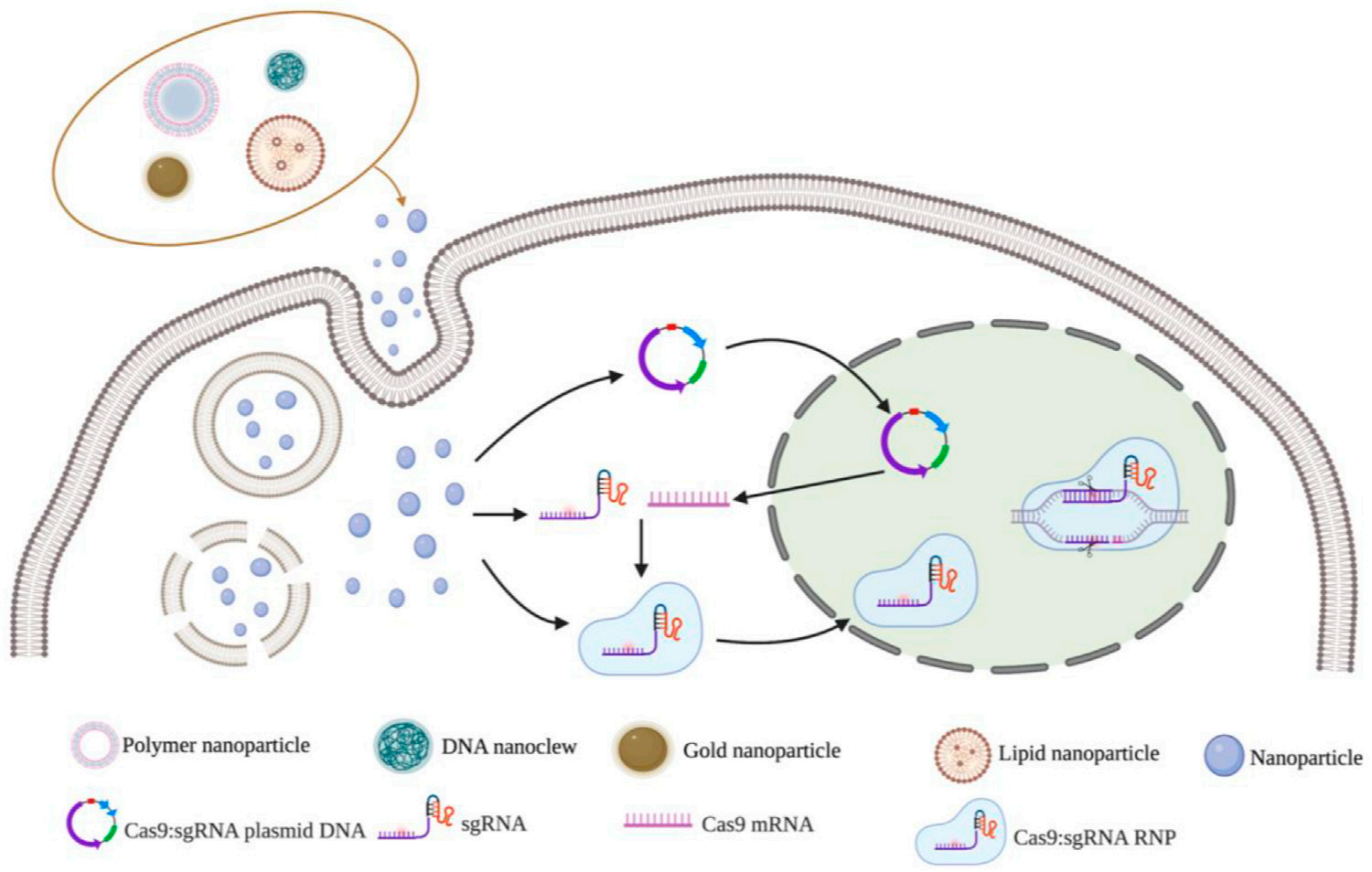
Nanoparticles vectors and gene editing strategy for CRISPR/Cas9 delivery system. Notes: Created with BioRender.com.

Non-viral vector delivery is an emerging research field. The principle of using a non-viral vector is to mediate the transport of CRISPR components by the physicochemical properties of synthetic or naturally occurring vectors. Currently, the non-viral vectors reported in the literature are mainly nanoparticles (Figure 3). and the nanomaterials commonly used to deliver CRISPR components include lipid nanoparticles, polymer nanoparticles, DNA nanoclew, inorganic nanoparticles (Table 4). These vectors are free of any viral components and can be used *in vitro* or *in vivo* as virus particles and help to improve safety and reduce immunogenicity without problems such as endogenous virus recombination.

**TABLE 4:** Non-viral vector delivery of CRISPR/Cas systems.

Approaches	Delivery strategies	CRISPR/Cas systems	Target cells/tissues	Target genes	References
LNPs	*in vitro, in vivo*	Cas9/sgRNA RNP	U2OS, inner ear	EGFP	Zuris et al. (2015)
	*in vitro, in vivo*	Cas9/sgRNA RNP	GFP-HEK, brain	GFP	Wang et al. (2016)
	*in vitro*	CRISPR/Cas9 plasmid	iPS	DNMT3B	Horii et al. (2013)
PEI	*in vitro*	Cas9 protein and sgRNA	MRSA	mecA	Kang et al. (2017)
BPEI-25K	*in vitro*	CRISPR/Cas9 plasmid	Neuro2	Slc26a4	Ryu et al. (2018)
PPC	*in vivo*	CRISPR/Cas9 plasmid	OS tissues	VEGFA	Liang et al. (2017)
PBA-modified polymer	*in vitro*	Cas9/sgRNA RNP	HeLa, NIH3T3, MDA-MB-231	AAVS1, HBB, CTNNB1	Liu et al. (2019)
Chitosan	*in vitro*	Cas9/sgRNA RNP	HeLa	-	Qiao et al. (2019)
NCL	*in vitro, in vivo*	Cas9/sgRNA RNP	U2OS	EGFP	Sun et al. (2015)
	*in vivo*	Cas12a/crRNA RNP	Blood, liver	Pcsk9	Sun et al. (2020)
AuNPs	*in vitro, in vivo*	Cas9/sgRNA RNP	hES, iPS, BMDCs	CXCR4, dystrophin	Lee et al. (2017)
	*in vivo*	Cas9/sgRNA RNP	Brain	mGluR5	Lee et al. (2018)
CPP	*in vitro*	Cas9/sgRNA RNP	hES, DF, HeLa, 293T, ECs	CCR5	Ramakrishna et al. (2014)
Exosomes	*ex vivo*	CRISPR/Cas9 plasmid	HSCs	HNF4α	Luo et al. (2021)
	*in vitro, ex vivo*	CRISPR/Cas9 plasmid	KPC689	Kras^G12D^	McAndrews et al. (2021)

Notes: iPS: pluripotent stem cells; LNPs: lipid nanoparticles; PEI: polyethyleneimine, PNPs: polymer nanoparticles; NCl: DNA, nanoclew; INPs: inorganic nanoparticles; CPP: cell-penetrating peptide; DNMT3B: methyl-CpG, binding protein 3b; MRSA: methicillin-resistant *staphylococcus aureus*; Slc26a4: solute carrier family 26 member 4; PPC: PEG-PEI-cholesterol; VEGFA: vascular endothelial growth factor A; OS: osteosarcoma; PBA: phenylboric acid; AAVS1: adeno-associated virus integration site 1; *HBB:* hemoglobin subunit beta*; CTNNB1:* catenin beta 1*;* EGFP: enhanced green fluorescent protein; AuNPs: gold nanoparticles; hES: human embryonic stem cells; induced pluripotent stem cells; BMDCs: bone marrow derived dendritic cells; CXCR4: C-X-C motif chemokine receptor 4; DF: dermal fibroblasts; ECs: embryonic carcinoma cells; CCR5: C-C motif chemokine receptor 5; HSCs: hematopoietic stem cells; HNF4α: hepatocyte nuclear factor 4.

*Lipid nanoparticles* (LNPs) are one of the most commonly used nucleic acid delivery systems and can be used to deliver RNA drugs, vaccines, or gene editing tools. The main principle of this delivery method is to combine negatively charged nucleic acid and positively charged liposome through electrostatic interaction to form lipid nanoparticles (Cong et al., 2013). Under the action of the external lipid layer, it helps the internal nucleic acid to cross the membrane into the target cell and avoids the degradation of RNA hydrolase and immune reaction. Felgner et al. first used liposomes to encapsulate and deliver DNA to mammalian cells in 1987 (Felgner et al., 1987). Currently, nucleic acid drugs using LNP have been approved, such as alnylam’s siRNA drug Onpattro, which proves the safety and effectiveness of this delivery system through the encapsulation efficiency is low.

Cationic lipids have been commercialized as a mature delivery vector for packaging negatively charged CRISPR plasmids, mRNA, sgRNA, or Cas9: sgRNA RNPs. Experimental results of many research teams have proved that lipid nanoparticles as CRISPR/Cas9 delivery vectors are expected to achieve gene therapy. For example, assembled RNPs were delivered by lipid nanoparticles to infect human cells *in vitro*, achieving up to 70%–80% genomic editing efficiency in cells (Zuris et al., 2015; Wang et al., 2016). The study of Horii et al. also demonstrated delivery of pDNA encoding Cas9 and sgRNA into human pluripotent stem cells using lipid nanoparticles successfully generated immune deficiency, centrosomal region instability, and facial abnormality syndrome (ICF) syndrome model with a transfection efficiency of 63% (Horii et al., 2013). An *in vivo* experiment, Cas9-mediated gene modification was achieved up to 20% in the bristle cells *via* using lipid nanoparticles (Zuris et al., 2015).

Functional modification of commercially available lipids can improve the transfection efficiency of liposomes, reduce the biological toxicity of liposomes, and better realize the targeting of CRISPR/Cas9 component delivery. Zhang et al. synthesized a novel delivery system for phospholipid-modified nanoparticles (PLNPs) modified with polyethylene glycol phospholipid. The system can concentrate and encapsulate pDNA encoding Cas9 and sgRNA (targeting PLK-1 gene) to form a core-shell structure (pLNP/DNA) (Zhang et al., 2017). Results showed that the transfection efficiency of A375 cells *in vitro* was about 47.4%. PLK-1 protein was significantly downregulated in mice and inhibited melanoma growth *in vivo* (>67%). Onuki et al. modified liposomes with 1, 2-dioleoyl-Sn-glycero-3-phosphocholine (DOPC), 1, 2-dipalmityl-Sn-glycero-3-phosphocholine (DPPC), and cholesterol for *in vitro* cell uptake in HeLa cells (Onuki et al., 2016). Experimental results showed that the modified liposomes had a higher rate of cell uptake. Rosenblum et al. used LNPs to package Cas9 mRNA and PLK1-targeting sgRNA, and injected CRISPR-LNP (cLNPs) into the brain of glioblastoma mice (Rosenblum et al., 2020). After detection, about 70% of the PLK1 gene was successfully edited in mice. It inhibited the growth of tumors and promoted apoptosis of tumor cells and increased the survival rate of mice by 30%. In addition, the authors decorated cLNP with antibodies overexpressing receptors on ovarian cancer cells. Results showed that the accumulation of EGFR-targeted cLNP in tumors was significantly higher than that of cLNP without antibody decoration.

The researchers also developed a phototriggered liposome delivery system that enables CRISPR/Cas9 gene editing to achieve spatial and temporal control with a high degree (Wang et al., 2018; Pan et al., 2019; Aksoy et al., 2020). For example, Alp Aksoy et al. loaded a phototrigger vitipofen into a liposome that could be activated by the light within 1 cm below the skin to release loaded CRISPR components (Aksoy et al., 2020). Experimental results showed that the fluorescence density of GFP decreased significantly after 6 min of illumination when phototriggered liposomes targeting the eGFP gene were microinjected into human HEK293 cells. Microinjection of phototriggered liposomes into zebrafish embryos resulted in a 77% knockout efficiency of the target gene. These results indicate that the modification of the lipid nanoparticle delivery system lays the foundation for the development of precise and controllable therapeutic methods.

*Polymer nanoparticles* are another widely used CRISPR component delivery carrier. Like lipid carriers, polymer carriers transport CRISPR components across membranes by endocytosis, and the polymer surfaces can easily be modified with additional targets, which allows them to deliver cargo to target tissues for controlled release of cargo. At present, the most used polymers are polyethyleneimine (PEI), polyamidoamine (PAMAM), and chitosan (CS). However, molecularly heavy polymers often exhibit greater cytotoxicity, thus these issues still need to concern about the safety, cost of production, efficacy over time, and gene size that must be solved to further enhance clinical applications. To adress this dilemma, researchers try to make great efforts by modifying polymers during intracellular delivery. A few modified or other polymers have demonstrated a promising ability to deliver mRNA/pDNA/RNP to disrupt targeting genes both *in vitro* and *in vivo*. Some examples are here. Kang et al. covalently conjugated PEI with Cas9 protein and then combined it with sgRNA of *mecA* gene to form polymer nanoparticles carrying CRISPR components (Kang et al., 2017). Results showed that the CRISPR system was successfully delivered to methicillin-resistant *staphylococcus aureus* (MRSA) by polymer nanoparticles, which effectively edited the target genome and was more efficient than the conventional lipid delivery system. Ryu et al. delivered CRISPR plasmid DNA into Neuro2 cells using a branched form of PEI (BPEI-25K) and successfully edited the genome at the Slc26a4 target site (Ryu et al., 2018). Liang et al. packaged plasmids containing vascular endothelial growth factor (VEGFA)-targeting sgRNA and Cas9 in PEG-PEI-cholesterol (PPC) lipid polymers and screened an osteosarcoma cell-specific ligand (LC09) to coat the surface of PPC lipid polymers (Liang et al., 2017). Results showed that LC09-packed vectors selectively distributed in both *in-situ* osteosarcoma and lung metastases reduced VEGFA expression and secretion, reduced angiogenesis, and inhibited tumor growth and metastasis without detectable toxicity. Liu et al. modified polyaminamine (PAMAM) with phenylboric acid (PBA) to form a PBA-modified polymer (P4), which ensures Cas9 protein can be delivered efficiently into various cell lines such as HeLa, NIH3T3, and MDA-MB-231 with high efficiency (Liu et al., 2019). In addition, in a study by Qiao et al., negatively charged red fluorescent protein (RFP) was encapsulated in CS to form RFP@CS NP to absorb ssDNA donors and Cas9 RNPs with negative charge. The modified nanoparticles enter the cytoplasm through the endocytosis pathway and release Cas9 RNPs and ssDNA donors to achieve efficient genome editing (Qiao et al., 2019).

*DNA nanoclew* Sun et al. developed a cocoon-like anticancer drug delivery system called DNA nanoclew (NCl) in 2014 (Sun et al., 2014). NCl, a spherical DNA structure assembled by long ssDNA, is highly biocompatible and can be degraded by DNase. Besides, it can customize easily due to the self-assembly. The researchers encapsulated DNase I in an acid-degraded polymer that was attached to the outside of the globular DNA. Under acidic biological conditions, DNase I is activated to degrade NCl and release the cargo encapsulated within the NCl. Researchers initially successfully delivered the anticancer drug doxorubicin (DOX) intracellular using NCl and induced drug release based on environmental conditions. In 2015, Sun et al. used NCl to deliver CRISPR/Cas9 RNPs. The Cas9 protein was fused with the nuclear localization signaling peptide, which helped CRISPR/Cas9 RNPs enter the nucleus (Sun et al., 2015). The cationic polymer PEI was coated on the NCl to induce the inner body to escape. Nanoparticles composed of Cas9/sgRNA/NC-12/PEI were finally formed. Experimental results showed that the delivery efficiency of CRISPR/Cas9 RNPs using NCl was 36% (Sun et al., 2015). It is worth mentioning that the same team demonstrated this carrier also can deliver Cas12a/CRISPR RNA (crRNA) RNP targeting *Pcsk9* gene to regulate serum cholesterol levels in 2020 (Sun et al., 2020).

*Inorganic nanoparticles* such as gold, iron, and silicon dioxide are also used to synthesize nanostructured materials for a variety of drug delivery and imaging applications. These inorganic nanomaterials are precisely formulated and can be designed in a variety of sizes, structures, and geometry. Gold nanoparticles (AuNPs) are the most well-studied and have been reported to deliver CRISPR/Cas9 RNPs. Unlike viruses or lipid or polymer carriers, gold nanoparticles are easy to control in size and distribution; also, unlike DNA nanocrystals, which rely on biomolecules as carriers, inert AuNPs do not trigger an immune response to the nanoparticles themselves (Lee et al., 2017). With this delivery system, researchers also got great efficacy in different diseases, even with toxicity and solubility limitations. For example, Lee et al. found correcting the mutant duchenne muscular dystrophy (DMD) gene to the wild type was corrected by 5.4% and the level of muscle fibrosis in DMD model mice was significantly reduced by using AuNPs carrier to deliver Cas9:sgRNA RNPs and donor DNA (Lee et al., 2017). Besides, an animal experiment showed the mGluR5 mRNA level in the brain of fragile X syndrome (FXS) model mice was reduced by 40-50%, and FXS behavioral phenotypes of mice were significantly alleviated by delivering the CRISPR-God complex knocking out *mGluR5* gene into the mouse brain (Lee et al., 2018).

Some researchers combine gold nanoparticles and liposomes to produce multi-targeted nanoparticle delivery systems. Firstly, gold nanoparticles were modified with TAT peptide, and then the modified gold nanoparticles were combined with Cas9 protein with nuclear localization signal, sgRNA targeting *Pcsk9* gene, and finally coated with lipid layer with galactose modification. A triple-targeted delivery system was developed in which galactose could target the non-sialic glycoprotein receptor on the surface of liver cells, enabling the composite nanoparticles to specifically target liver cells. Experimental results showed that the composite nanoparticles could effectively reduce plasma *Pcsk9* level and LDL-C level in mice by injection into the tail vein, and no off-target effect was detected (Zhang et al., 2019).

*Other non-viral vector delivery methods* Similar to liposome nanoparticles, cell-penetrating peptide (CPP) is also a traditional drug delivery carrier. It is cationic and can be fused with the cell membrane through electrostatic interaction and enucleated into the cell. Liposome nanoparticles are commonly used to deliver nucleic acids, while CPPs are commonly used to deliver fusion proteins. Ramakrishna et al. bind Cas9 protein to CPP through thioether bond, and then bind sgRNA to form positively charged nanoparticles (Ramakrishna et al., 2014). The delivery of the CPP-Cas9: sgRNA complex into a variety of human cells resulted in effective gene destruction in cells with a lower off-target effect than plasmid transfection. In addition, exosomes are a natural and biocompatible mean of cell-to-cell communication. Exosomes can be transferred to neighboring and distant cells by diffusion and systemic circulation, mediating cell to cell communication. Exosomes are also being considered as potential nature delivery technologies for CRISPR components. The study of Luo et al. and other researchers put this theory into practice, Luo et al. first purified exosomes from culture supernatant, then loaded CRISPR/Cas9 plasmids into exosomes using the commercially available exosome transfection reagent, and finally incubated the modified exosomes and cells *in vitro* and injected these exosomes into mouse *via* tail vein. Results showed exosomes effectively encapsulated and delivered the CRISPR/dCas9-VP64 system into hematopoietic stem cells (HSCs) *in vitro* and *in vivo* (Usman et al., 2018; Luo et al., 2021)*.* McAndrews et al. used a similar method to successfully knock out the mutant *Kras* ^*G12D*^ oncogenic allele in pancreatic cancer cells, and cell proliferation and tumor growth were inhibited (McAndrews et al., 2021). Although exosomes as nanocarriers have shown many advantages such as low immunogenicity, high biocompatibility, crossing biological barriers, combination therapy, the risk of DNA fragment integration during genome editing and the large-scale production of exosomes for clinical application should be concerned (Duan et al., 2021).

Zhang et al. developed poly (ethylene glycol) methyl ether-block-poly (lactide-co-glycolide) (PEG-b-PLGA) copolymer-based nanoparticle formulated with polyethyleneimine to target endothelium for robust genome editing. Results showed protein expression selectively decreased about 80% in endothelial cells (Zhang et al., 2022). Additionally, Wang et al. designed NIR-responsive biomimetic nanoparticles (UCNPs-Cas9@CM) that could effectively deliver Cas9 RNP to achieve effective genome editing for HBV therapy. In HBV-infected cells, the expression of HBsAg, HBeAg, HBV pgRNA and HBV DNA along with cccDNA was inhibited (Wang et al., 2022).

## Application of CRISPR/Cas9 in diseases

Many human diseases are caused by gene mutations, including defects or abnormal expression of specific genes in the genome. By ‘correcting’ the mutated genes, the corresponding diseases can be completely cured, named gene therapy. As a simple and efficient gene editing technology, CRISPR/Cas9 has been reported to be used in gene therapy for genetic diseases such as monogenic diseases, infectious diseases, tumor and other diseases caused by gene mutation, showing good application prospects in the field of disease treatment. To date, CRISPR/Cas9 systems have also been applied for clinical therapy (Table 5).

**TABLE 5:** Clinical trials of gene therapy using CRISPR/Cas technology.

	Diseases	Target genes	Target cells/viruses	CRISPR/Cas systems	Stages	Companies/institutes	Country	Clinicaltrials. Gov ID
1	HPV-related cervical intraepithelial neoplasiaⅠ	E6/E7T1	HPV16	CRISPR/Cas9 plasmid	Phase 1	First Affiliated Hospital, Sun Yat-Sen University	China	NCT03057912
2	Severe sepsis	-	-	CRISPR/Cas12a	Not Applicable	The Affliated Drum Tower Hospital, Medical School of Nanjing University	China	NCT04178382
3	Refractory viral keratitis	-	HSV-1	CRISPR/Cas9 mRNA	Not Applicable	Eye & Ent Hospital of Fudan University	China	NCT04560790
4	COVID-19 respiratory infection	PD-1and ACE2	T cells	CRISPR/Cas9 plasmid	Phase 1/2	Mahmoud Ramadan mohamed Elkazzaz, Kafrelsheikh University	Egypt	NCT04990557
5	Severe pneumonia	-	-	CRISPR/Cas12a	Not Applicable	Chinese Medical Association	China	NCT05143593
6	Thalassemia	HBB	iHSCs	CRISPR/Cas9	Early Phase 1	Allife Medical Science and Technology Co., Ltd	China	NCT03728322
7	Gastro-intestinal (GI) cancer	CISH	T cells	CRISPR/Cas9	Phase 1/2	Masonic *Cancer* Center, University of Minnesota	United States	NCT04426669
8	NF1	NF1	iPSC	CRISPR/Cas9	Suspended	Roger Packer, Children’s National Research Institute	United States	NCT03332030
9	Sickle cell disease	Hemoglobin genes	RBCs	CRISPR/Cas9 RNPs	Phase 1/2	University of California, Los Angeles & Berkeley	United States	NCT04774536
10	Kabuki syndrome 1	KMT2D	MSCs	CRISPR/Cas9	-	Association Française contre les Myopathies Telethon	France	NCT03855631
11	Advanced EGFR-positive solid tumors	TGFβRⅡ	CART cells	CRISPR/Cas9	Phase 1	Chinese People’s Liberation Army General Hospital	China	NCT04976218
12	Relapsed/sefractory CD5^+^ hematopoietic malignancies	CD5	CT125A cells	CRISPR/Cas9	Early Phase 1	Huazhong University of Science and Technology	China	NCT04767308
13	Acute myeloid leukemia	CD33	HSCs	CRISPR/Cas9	-	Vor Biopharma, Inc	United States	NCT05309733
14	Acute myeloid leukemia	WT1	TCR T cells	CRISPR/Cas9	Phase 1/2	Intellia Therapeutics, Inc	United States	NCT05066165
15	Hereditary angioedema	KLKB1	Hepatocytes	CRISPR/Cas9	Phase 1/2	Intellia Therapeutics, Inc	United States	NCT05120830
16	Mesothelin positive multiple solid tumors	PD-1, TCR	CART cells	CRISPR/Cas9	Phase 1	Chinese People’s Liberation Army General Hospital	China	NCT03545815
17	β-Thalassemia	BCL11A	HSPCs	CRISPR/Cas9 RNPs	Phase 2/3	Vertex Pharmaceuticals Incorporated	United States	NCT03655678
18	β-Thalassemia, Sickle cell disease	BCL11A	HSPCs	CRISPR/Cas9 RNPs	Phase 3	Vertex Pharmaceuticals Incorporated	United States	NCT05477563
19	Multiple myeloma	BCMA	T Cells	CRISPR/Cas9	Phase 1	CRISPR Therapeutics	United States	NCT04244656
20	Sickle cell disease, hematological diseases	BCL11A	HSPCs	CRISPR/Cas9 RNPs	Phase 2/3	Vertex Pharmaceuticals Incorporated	United States	NCT03745287
21	Sickle cell disease	BCL11A	HSPCs	CRISPR/Cas9 RNPs	-	National Human Genome Research Institute	United States	NCT03167450
22	Renal cell carcinoma	CD70	T Cells	CRISPR/Cas9	Phase 1	CRISPR Therapeutics	United States	NCT04438083
23	Transfusion dependent β-Thalassaemia	BCL11A	HSPCs	CRISPR/Cas9 RNPs	Phase 1	EdiGene (GuangZhou) Inc	China	NCT04925206
24	B-cell malignancies	CD19	T Cells	Cas9 protein and sgRNA	Phase 1	CRISPR Therapeutics	United States	NCT04035434
25	T or B cell malignancies	CD70	T Cells	CRISPR/Cas9	Phase 1	CRISPR Therapeutics	United States	NCT04502446
26	Mesothelin positive multiple solid tumors	PD-1	CAR-T Cells	CRISPR/Cas9	Phase 1	Chinese People’s Liberation Army General Hospital	China	NCT03747965
27	Leukemia, lymphoma	TCR, B2M	CAR-T Cells	CRISPR/Cas9	Phase 1/2	Chinese People’s Liberation Army General Hospital	China	NCT03166878
28	β-Thalassemia, hematologic diseases, hemoglobinopathies	BCL11A	HSPCs	CRISPR/Cas9	Phase 3	CRISPR Therapeutics	United States	NCT05356195
29	Sickle cell disease, hydroxyurea failure, hydroxyurea intolerance	BCL11A	HSPCs	CRISPR/Cas9	Phase 3	CRISPR Therapeutics	United States	NCT05329649
30	β-Thalassemia, sickle cell disease, hematologic diseases, hemoglobinopathies, sickle cell anemia	BCL11A	HSPCs	CRISPR/Cas9	-	CRISPR Therapeutics	United States	NCT04208529
31	Leukemia, lymphoma	CD19, CD20, CD22	CAR-T Cells	CRISPR/Cas9	Phase 1/2	Chinese People’s Liberation Army General Hospital	China	NCT03398967
32	Esophageal cancer	PD-1	T Cells	CRISPR/Cas9	Not Applicable	Hangzhou *Cancer* Hospital	China	NCT03081715
33	Diabetes mellitus type 1, glucose metabolism disorders, metabolic disease, endocrine system diseases, autoimmune diseases, immune system diseases	-	PEC210A cells	CRISPR/Cas9	Phase 1	CRISPR Therapeutics	United States	NCT05210530
34	B-cell acute lymphoblastic leukaemia	CD52, TRAC	T cells	CRISPR/Cas9	Phase 1	University College London	UK	NCT04557436
35	Advanced Hepatocellular Carcinoma	PD-1	T cells	CRISPR/Cas9	Phase 1	Central South University	China	NCT04417764
36	Metastatic NSCLC	PD-1	T cells	CRISPR/Cas9	Phase 1	Sichuan University	China	NCT02793856
37	LCA10, eye diseases, retinal degeneration	CEP290	-	CRISPR/Cas9	Phase 1/2	Editas Medicine, Inc	United States	NCT03872479
38	HIV-1-infection	CCR5	HSCs	CRISPR/Cas9	Not Applicable	Affiliated Hospital to Academy of Military Medical Sciences	China	NCT03164135

Notes: HPV: human papillomavirus; HSV-1: herpes simplex virus type I; PD-1: programmed death-1; ACE2: angiotensin converting enzyme-2; CCR5: C-C motif chemokine receptor 5; HIV: human immunodeficiency virus; iHSCs: induced hematopoietic stem cells; HBB: hemoglobin subunit beta; CISH: cytokine-induced SH2 protein; NF1: neurofibromatosis type 1; HSPCs: hematopoietic stem progenitor cells; RBCs: red blood cells; MSCs: mesenchymal stem cells; KMT2D: lysine methyltransferase 2D; CART: chimeric antigen receptor modified T; TGFβR Ⅱ: transforming growth factor-β receptor Ⅱ; CT125A cells: a novel CAR T cell; HSCs: hematopoietic stem cells; TCR: T cell receptors; WT1: wilms’ tumor gene 1; KLKB1: kallikrein B1; BCL11A: transcription factor B-cell lymphoma/leukemia 11A; HSPCs: hematopoietic stem and progenitor cells; BCMA: B cell maturation antigen; B2M: Beta-2-microglobulin; PEC210A: allogeneic pancreatic endoderm cells; TRAC: T cell receptor alpha constant; NSCLC: non-small cell lung cancer; LCA10: leber congenital amaurosis type 10; CEP290: centrosomal protein 29.

### Monogenic diseases

β-mediterranean anemia (TDT) and sickle cell disease (SCD) are inherited blood disorders caused by mutations in the β globin gene. Currently, the only treatment for these diseases is allogeneic stem cell transplantation, but this approach is limited and some complications may occur such as transplant conditioning, graft *versus* host disease (GVHD), and graft rejection (Leonard and Tisdale, 2018). Another potential therapeutic strategy for these diseases is gene therapy targeting autologous HSCs through gene addition or gene modification. At present, one method of gene therapy for TDT and SCD is to replace defective and insufficient adult hemoglobin (HbA) with fetal hemoglobin (HbF). However, due to the binding site of the repressor at the HbF promoter HBG1, HbF expression is turned off around 1 year of age. Humbert et al. used CRISPR/Cas9 system with RNPs form to knockout BCL11A gene that inhibited HbF expression at HBG1 of CD34^+^ HSCs and progenitor cells, and then re-injected the cells into non-human primate models, and found that the transplantation rate of gene-edited cells was as high as 30%, lasting more than 1 year (Humbert et al., 2019). HbF was expressed in up to 18% of erythrocytes in peripheral blood, demonstrating that the edited cells effectively and stably reactivated HbF expression, a result sufficient to reverse the symptoms of sickle cell anaemia and TDT. CTX001 therapy has been developed for TDT and SCD, in which patients’ CD34^+^ cells are edited *in vitro* using CRISPR/Cas9 RNPs to disrupt the expression of the BCL11A gene and then transfused back into the patient. Clinical results showed that total HbA and HbF increased significantly in 15 patients with β -thalassemia and seven patients with sickle cell disease after treatment (Frangoul et al., 2020).

DMD is a genetic disorder caused by mutations in the DMD gene, which encodes a protein necessary for muscle contraction. People with this disease will show progressive muscular dystrophy in childhood, and there is currently no effective treatment for DMD. Long et al. showed that the CRISPR technique was used to perform *in vitro* gene editing on the zygotes of DMD mutated mice. The zygotes were injected with Cas9, sgRNA, and HDR templates targeting DMD, and then transplanted into pseudopregnancy mice (Long et al., 2014). DMD protein expression was restored in the offspring mice and the mice had normal skeletal muscle function. Xu et al. modified the adenine base editor (ABE) by fusing ABE with Cas9 protein and packaging them in AAV9 virus and injecting them into Duchenne syndrome mice *via* tail vein (Xu et al., 2021). After 10 months of AAV9 injection, functional analysis showed that myocardial fibrosis was significantly reduced in DMD mice, and muscle contraction function was enhanced, without significant toxic and side effects. This study achieved efficient and precise repair of DMD gene mutations in adult mice. Although these studies cannot be applied to humans at present, they provide important preclinical guidance for the treatment of DMD and offer new hope for the treatment of muscular dystrophy in the future.

Leber congenital amaurosis type 10 (LCA10), the most common type of LCA, is an autosomal recessive disorder caused by a mutation in the biallelic gene CEP290. CEP290 protein deficiency leads to impaired function of retinal photoreceptor cells, usually in early infancy, and patients present with severe cone malnutrition and low vision, or even complete loss (Chang et al., 2006). There are no drugs available to treat LCA10. EDIT-10 developed a CRISPR/Cas9 gene editing treatment for LCA10 by Editas Medicine, which consisted of two gRNAs that bound with the mutation to each end of the intron. The Cas9 protein was induced to shear at both ends of the intron, after which the cell repaired the DNA sequence through NHEJ, and the repaired gene sequence could be expressed normally, producing functional CEP290 protein, which restored the function of photoreceptor cells. In preclinical experiments in mice and non-human primates, EDIT-101 was delivered *via* a subretinal injection to the subretinal lumen (Ruan et al., 2017) (Maeder et al., 2019). The results showed that EDIT-101 restored normal expression of CEP290 and demonstrated the ability of CRISPR/Cas9 to edit somatic cells *in vivo*. Supported by these results, Editas Medicine initiated a Phase I/II clinical trial of EDIT-101 for LCA10 in July 2019, in which 2 of 3 treated patients showed improved visual function and clinical activity signals on photosensitivity tests, and participants in the trial were well tolerated by the investigational gene editing treatment (Maeder et al., 2019) (NCT03872479).

Huntington’s disease (HD) is an inherited neurological disorder caused by autosomal dominant mutations, often presenting with dance-like motor symptoms, accompanied by psychiatric symptoms and cognitive decline. The disease is caused by abnormal duplication of CAG in a specific DNA sequence within the Huntington gene. The higher the copy number, the earlier the disease manifests. The research team of Seiya Oura et al. engineered Cas9 from *Streptococcus* pyogenes and modified spCas9 can recognize NGN PAM. By targeting the boundaries of CAG repeats with spCas9-NG, the repeated bundles in embryonic stem cells derived from HD mice were accurately contracted. The repaired mice returned to the normal phenotype (Oura et al., 2021).

### Infectious diseases

Viral infection can also cause malignant diseases and cancers, such as hepatocellular carcinoma caused by HBV infection, cervical cancer caused by human papillomavirus (HPV) infection, and HIV infection caused by AIDS. HBV infection can cause cirrhosis and hepatocellular carcinoma. Previous treatments for chronic infection caused by HBV have been to use nucleoside analogues to inhibit HBV DNA synthesis in liver cells but have no effect on covalently closed circular DNA (cccDNA), the template for HBV RNA transcription. Therefore, the disease caused by HBV infection cannot be completely cured. Some researchers proposed that the CRISPR/Cas9 system could be used to directly target HBV cccDNA and inhibit HBV replication (Kennedy et al., 2015; Seeger and Sohn, 2016). Dong et al. designed four sgRNAs targeting HBV conserved regions, and results at the cellular level showed that the expression of sgRNAs and Cas9 reduced virus production in Huh7 cells and HepG2 cells (Dong et al., 2015). They further introduced the sgRNA-Cas9 plasmid into a mouse model of HBV infection by tail vein injection. Results showed that cccDNA and HBV protein levels in mice were significantly reduced. It provides a new therapeutic strategy for chronic HBV infection. There are two important oncogenes on the genome of the HPV virus that cause cervical cancer, the E6 gene and the E7 gene. The expression of these two genes can induce the degradation of tumor suppressor factors p53 and Rb1 and promote the progress of malignant cervical cancer. It has been proved that the targeted destruction of E6 and E7 genes in HPV-induced cervical cancer cells by using the CRISPR/Cas9 system can lead to the inactivation mutation of E6 and E7 genes, which can induce cancer cell cycle arrest and apoptosis. Therefore, the use of viral vectors to deliver the CRISPR/Cas9 system targeting E6 and E7 to tumor cells is considered to be an effective way to treat HPV infection-related diseases (Kennedy et al., 2014; Zhen et al., 2016). In addition to cutting the virus genes themselves, the CRISPR/Cas9 system can also target coreceptors in the body necessary for virus replication to reduce virus production.

Acquired immunodeficiency syndrome (AIDS) also is an infectious disease caused by human immunodeficiency virus (HIV). In the process of HIV infection, the CCR5 membrane protein is one of the main coreceptors of HIV-1 to invade the body cells. An inactivated mutation of the CCR5 protein prevents HIV-1 integration in the body. Based on this theory, Xu et al. knocked out the CCR5 gene of donor CD34^+^ adult HSCs by CRISPR/Cas9 technology and transplanted them into patients with AIDS-associated leukemia (Xu L. et al., 2019). In the follow-up of 19 months after treatment, CCR5 gene editing was continuously detected in bone marrow cells. The efficiency was 5.20%–8.28%, and the patient’s leukemia received sustained remission. However, it was less effective against HIV, with the serum viral load increasing from undetectable levels to 3×10^7^/ml when anti-HIV drugs were suspended for 4 weeks. This is the first time in the world that the CCR5 gene of human HSCs was knocked out by genome editing technology and then transfected back into AIDS patients with leukemia. The data of this study showed that the effect of HIV resistance was not good, but the use of genome editing technology has important guiding significance in the treatment of AIDS.

### Tumor

During tumorigenesis, T cell-mediated immune responses kill tumor cells. Some immune checkpoints (such as PD-1 and CTLA4) distinguish normal cells from cancer cells by recognizing their ligands on the cell surface, but some tumor cells evade the immune system by producing the ligands of immune checkpoints such as PD-L1 (Zhou et al., 2017). In addition to restoring the ability of the immune system to kill tumors by developing drugs that interrupt immune checkpoints (Topalian et al., 2015; Hoos, 2016). Some researchers have proposed that the CRISPR/Cas9 system can be used to modify differentiated T cells in patients to target the expression of PD-1 on T cells, so that CD4 T cells can be re-targeted and destroy cancer cells (Schumann et al., 2015). In a phase I clinical trial of immunotherapy in humans, three subjects were enrolled, two with multiple myeloma and one with refractory metastatic sarcoma that had not responded to previous therapy. We used CRISPR/Cas9 to remove two genes encoding endogenous TCR, TCRα and TCRβ, and PDCD1 encoding PD-1 in T cells, and inserted a synthetic cancer-specific TCR gene (NY-ESO-1) into T cells to identify tumor cells. The edited T cells were then transfused back into the patient. Clinical results showed that patients who received the treatment showed tumor regression, which lasted for 4 months, proving that this treatment is feasible (Stadtmauer et al., 2020). In a Phase I trial of immunotherapy using CRISPR/Cas9-mediated PD-1-edited T cells in patients with advanced NSCLC (NCT02793856), T lymphocytes were extracted from patients with lung cancer, Then, Cas9 and sgRNA plasmids were co-transfected into T cells by electroporation, and the PD-1 gene of these T cells was targeted and deleted. The gene edited T cells were amplified *in vitro* and re-transfected into patients. Clinical trials showed that the gene-edited T cells remained in the patient’s blood for at least 4 weeks, indicating that the treatment was safe and durable (Lu et al., 2020). Taken together, these studies provide preliminary evidence for the feasibility of multiple gene editing using CRISPR/Cas9 at a clinical level, but more clinical trials are needed to demonstrate or improve this treatment.

The above studies indicate that CRISPR/Cas9-based genome editing technology can provide a potential method to cure some genetic diseases, cancer, and other diseases, suggesting a new treatment strategy for patients different from traditional treatment methods.

## Limitation and perspective of CRISPR/Cas system

To date, CRISPR-based technologies have become popular and powerful genome editing methods to inhibit/activate genes by various types of CRISPR systems like CRISPR, CRISPRi, and CRISPRa. With the advancement of CRISPR systems, genetic disease treatment has been improved by intervening in genetic, epigenetic, and transcriptional aberrations. This potent technology has been used in clinics to treat some genetic diseases like SCD, HD, TDT, and DMD, even though it is in the early stages of study. However, there are still many problems with the CRISPR/Cas system and delivery methods, which hinder its advancement and clinical application, such as the off-target effect, the efficiency of the DNA repair mechanisms, and how to deliver the CRISPR/Cas system safely and efficiently to the target location.

### Off-target effect

The off-target effect has become the main safety issue in CRISPR/Cas9-based therapeutic genome editing. When using the CRISPR/Cas system for gene editing, it is necessary to design gRNA of about 20bp targeted sequence to guide Cas9 to cut the gene. Nonetheless, the practice has proved that this gRNA is not absolutely specific, and it may recognize other gene sequences similar to the target sequence on the genome, resulting in off-target cutting and mutation of other non-target genes. Many approaches have been reported to reduce the off-target effect, such as selecting sites with fewer off-target sequences when designing gRNA. Hsu et al. proposed that reducing the content of Cas9 protein in the system could significantly reduce the off-target effect, but it would also lead to low efficiency of targeted cutting (Hsu et al., 2013). Other research groups have proposed that Cas9 can be modified to reduce off-target effects by designing two different mutant forms of Cas9 (one inactivates HNH nuclease activity, the other Cas9 protein inactivates RuvC nuclease activity), and the two treated Cas9 proteins are respectively expressed by fusion with the positive or negative gRNA of the targeted site. Only after the two Cas9 proteins are respectively guided by gRNA at specific sites, can double chain cleavage be performed to cause DSB. On the contrary, off-target bonding can only result in single-strand cutting and can be repaired quickly (Ran et al., 2013a; Mali et al., 2013; Cho et al., 2014; Shen et al., 2014). Moreover, researchers also designed dCas9-FokI fusion protein and forward and reverse gRNA binding to specific sites to achieve targeted cutting with low miss effect according to the fact that FokI nuclease monomer cannot play a role and only the dimerization state can give play to the characteristics of enzyme activity (Guilinger et al., 2014; Tsai et al., 2014). What’s more, Bravo et al. observed the complex structure of Cas9 formed with mismatched DNA during the cutting process using Cryo-EM (Bravo et al., 2022). It was found that in the case of mismatch, a ring in the RuvC domain of Cas9 protein was structurally altered, which stabilized Cas9 and activated it. Therefore, they mutated all seven stable residues into aspartic acid by changing the stable residues in the molecular structure of the Cas9 protein to generate a new SuperFi-Cas9, which reduced the incidence of off-target cuts. The off-target effect might be resolved through continuous enhancement of the specificity of gRNA and Cas9 for site recognition and a series of structural modifications of Cas9 protein.

### DNA repair efficiency

Another limiting factor is the efficiency of the DNA repair mechanisms. Precise gene insertion, deletion or base replacement during gene editing using the CRISPR/Cas9 system relies on HDR. Nevertheless, studies have demonstrated that DSB repair in mice is more favorable to NHEJ even in the presence of donor template DNA (Maruyama et al., 2015). Therefore, it is necessary to improve the efficiency of HDR repair and inhibit NHEJ-mediated repair. To date, selectively disrupt NHEJ, or directly boost the HDR repair pathway is the common approaches to the efficiency of HDR repair in the CRISPR/Cas9 system, which has proven successful. For example, SCR7, NU7441, and KU-0060648, small molecule inhibitors, which can interfere with the binding of DNA ligase IV to DNA and blocks terminal ligand, resulting in NHEJ inhibition (Srivastava et al., 2012; Maruyama et al., 2015; Robert et al., 2015). Some research teams proposed constructing corresponding gene-edited cell lines by using NHEJ-deficient cell lines (Weinstock and Jasin, 2006) or by silencing NHEJ-related genes (Chu et al., 2015). Besides, RAD51 activity enhanced by RS-1 involved strand exchange and the search for homology. Thus, researchers applied RS-1 to increase Cas9-stimulated HDR in zebrafish embryos and hPSCs (Zhang et al., 2018; Jayavaradhan et al., 2019). Since HDR mainly occurs in the S and G2 phases of the cell cycle (Heyer et al., 2010), cell cycle synchronization can be performed with CRISPR/Cas9 system to improve the repair efficiency of HDR (Lin et al., 2014). Alternatively, modified Cas12a proteins can be used, and the cleaved sticky ends of Cas12a increase the efficiency of HDR-mediated insertion compared to the blunt ends of Cas9. These existing reports can assist us to improve the efficiency of homologous recombination to achieve precise gene insertion, deletion, or base replacement. Nevertheless, the application of these strategies relies on some factors such as the type of cell, species, the location of gene, and experimental design. Moreover, safety problems like toxicity may increase when using the suppression of NHEJ-relevant factors both *in vitro* and *in vivo*. Relatively, these methods including favor HDR factors, timely delivery of Cas9, and all-in-one strategies might have greater applicability. Therefore, enhancing HDR frequencies by combining various approaches is more likely to be the optimal choice in the future.

### Accuracy of CRISPR/Cas delivery system

How to deliver CRISPR/Cas systems safely and efficiently to the target location is the main limitation in clinical application. The transfection efficiency of the virus vector is high, but it is easy to cause non-target insertion mutagenesis, high immunogenicity, and poor packaging ability caused by the small size of the virus. And the application scenarios are limited. There are also many problems to be solved during the delivery of non-viral vectors in the CRISPR/Cas9 system, such as how to avoid the recognition and clearance of the reticuloendothelial system and how to target specific tissues. It is necessary for researchers to carry out different ligand modifications or structural modifications of non-viral vectors, so as to enhance the targeting of vectors, promote cell uptake and improve delivery efficiency. The safety of non-viral delivery methods also needs to be addressed. Currently, the final location of various components in the non-viral nanoparticle delivery system is unknown, and the duration of their residence in the body, and whether they have long-term toxicity remain to be resolved; In the process of CPP delivery, the carrier is easily trapped into the endosome after entering the cell and finally digested by protease. When exosomes are used as delivery vectors, the transfer range of exosomes is not controllable, and exosomes are difficult to prepare. The existing delivery methods have their own advantages and disadvantages. Thus, delivery systems need to be considered wisely and carefully before exercising delivery. In addition to choosing the delivery methods carefully, actively seeking new delivery methods, continuously optimizing existing non-viral delivery vectors with high safety and targeting also need to be observed and developed.

## Conclusion

Since CRISPR/Cas technology was discovered, it has revolutionized the field of biology, biomedicine, and even agriculture. With the strengthening of this technology, it is becoming easier and easier to precisely edit genes in eukaryotes and prokaryotes, and curing untreated diseases is also possible. Apart from the problems mentioned above, ethical issues need further discussion when this technology is applied for editing human embryos to treat diseases. There is no denying that CRISPR technology has a bright future ahead, but it must be transformed to the next level by addressing the challenges discussed above at the earliest.
